# Peritoneal Recurrence of Squamous Cell Carcinoma in a Young Woman After Conization for Microinvasive Cervical Cancer

**DOI:** 10.7759/cureus.54091

**Published:** 2024-02-12

**Authors:** Isao Otsuka

**Affiliations:** 1 Department of Obstetrics and Gynecology, Kameda Medical Center, Kamogawa, JPN

**Keywords:** chemoradiation, transtubal spread, peritoneal recurrence, conization, microinvasive carcinoma, squamous cell carcinoma, cervical cancer

## Abstract

Microinvasive squamous cell carcinoma of the cervix develops mainly in young women. As metastases rarely occur, cervical conization to preserve fertility is often performed. We report a case of peritoneal recurrence developed after conization. A 31-year-old nulligravid woman with microinvasive squamous cell carcinoma of the cervix was treated with laser conization. Pathology showed a stromal invasion of <1 mm and a longitudinal spread of 3 mm without lymphovascular space involvement. Forty-seven months after conization, a pelvic examination revealed a firm, immobile mass on the right side of the pelvis. Transvaginal ultrasonography and magnetic resonance imaging showed a 3.8-cm solid mass located right of the rectum and anterior to the sacrum. A fine-needle biopsy showed squamous cell carcinoma. The tumor was diagnosed as a metastasis of cervical carcinoma. After salvage concurrent chemoradiation, the patient was well and had no evidence of disease at 90 months after the treatment. In this case, tumor cells appear to spread through the endometrial cavity and the lumen of the fallopian tube.

## Introduction

Cervical cancer is the fourth most commonly diagnosed cancer in women worldwide [1]. In young women with microinvasive squamous cell carcinoma of the cervix, i.e., a depth of invasion of 3 mm or less, who desire to preserve fertility, cervical conization is an effective and safe treatment option [2]. Although metastasis rarely develops in these patients, pelvic lymph node metastases have been reported [3-6]. However, peritoneal recurrence after conization has not been reported in the literature. We report herein a case of microinvasive squamous cell carcinoma of the cervix that was treated with conization but subsequently developed peritoneal recurrence without lymphadenopathy.

## Case presentation

**Figure 1:**
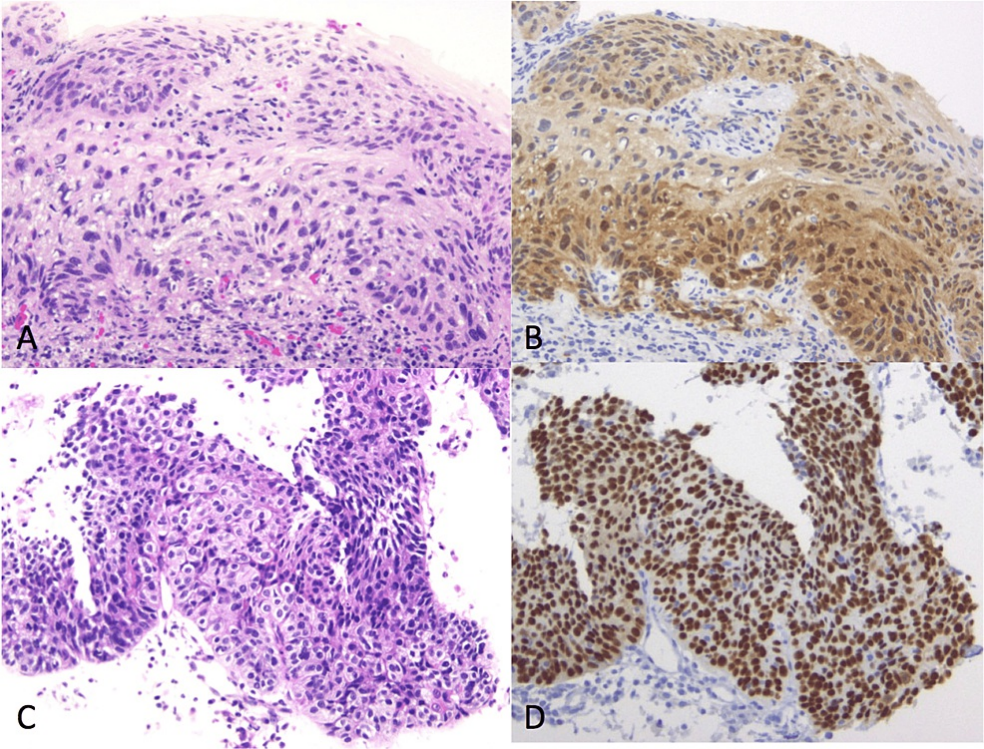
A Conization specimen (hematoxylin–eosin), B. Immunohistochemistry with p16. Microinvasive foci are seen. C. Biopsy of the tumor shows a squamous cell carcinoma (hematoxylin–eosin). D. Immunohistochemistry with p16. Tumor cells are diffusely positive.

A 31-year-old nulligravid woman was referred to our clinic because of an abnormal pap smear (HSIL). She had experienced dysmenorrhea and hypermenorrhea since menarche at 15 years old but did not report any symptoms associated with cervical lesions. Two biopsies of the cervix that were performed under colposcopy showed microinvasive squamous cell carcinoma in one sample and squamous cell carcinoma in situ in the other. Transvaginal ultrasonography revealed a normal retroverted uterus and normal bilateral ovaries. Then, she underwent excisional laser conization of the cervix. The pathology of the conization specimen showed microinvasive squamous cell carcinoma with a stromal invasion of <1 mm and a longitudinal spread of 3 mm without lymphovascular space involvement (Figures 1A, 1B).﻿

**Figure 2:**
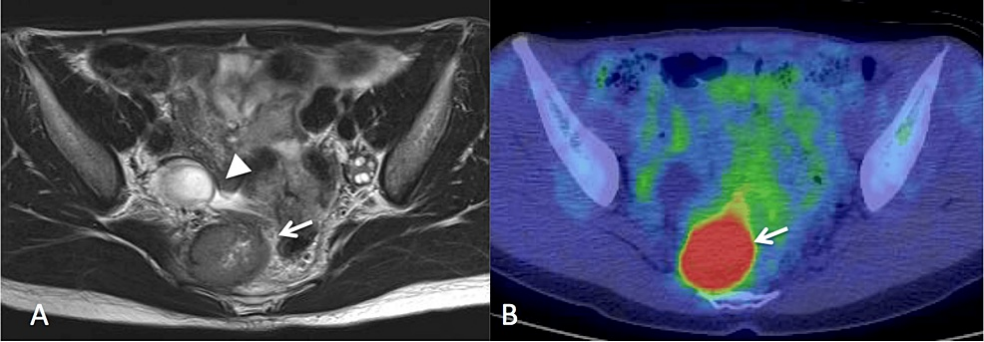
A. Axial view of a T2-weighted magnetic resonance image. Tumor (arrow) and normal right ovary (arrowhead) are observed; B. Positron emission tomoguraphy/computed tomography. An abnormal uptake of [18F]fluoro-deoxy-D-glucose (SUVmax of 20.8) is observed.

The patient was diagnosed with stage IA1 cervical cancer. CIN3 was observed on the endocervical margin of the conization specimen, and atypical epithelium was observed in the endocervical curettage specimen, which suggests that CIN might exist in the residual endocervix. As she wished to preserve fertility, a subsequent hysterectomy was not performed, and she was followed up with a pap smear with transvaginal ultrasound, conducted every three months in the first and second years and every four months in the third and fourth years. Positron emission tomography-computed tomography (PET/CT) imaging performed 12 and 35 months after conization showed no abnormalities. Although pap smears had remained negative, a pelvic examination at the outpatient visit 47 months after conization revealed a firm, immobile mass on the right side of the pelvis. Transvaginal ultrasonography revealed a 3.8-cm solid mass adjacent to the normal right ovary. Magnetic resonance imaging showed a solid mass located right of the rectum and anterior to the sacrum (Figure 2A).﻿

PET/CT showed an abnormal [18F]fluoro-deoxy-D-glucose uptake in this tumor (Figure 2B) with no other abnormal findings. Her serum squamous cell carcinoma antigen was slightly elevated, 3.0 ng/mL. A fine needle aspiration biopsy under colonoscopy showed squamous cell carcinoma (Figures 1C, 1D). The tumor was diagnosed as metastasis from cervical squamous cell carcinoma treated four years earlier. As the tumor was considered to be difficult to resect completely by surgery, concurrent chemoradiation was performed. After the completion of chemoradiation with 50.4 Gy of external beam radiation and five cycles of cisplatin (40 mg/m2), the solid mass disappeared. She was well and had no evidence of disease 90 months after the completion of the salvage therapy. 

## Discussion

This case indicates that microinvasive squamous cell carcinoma of the cervix can spread into the pelvic peritoneal cavity and develop a recurrent tumor without a clinically detectable lymph node or ovarian metastasis. In this case, tumor cells may spread through the endometrial cavity and the lumen of the fallopian tube.

Four possible pathways of spread for cervical carcinoma are: 1) direct extension, 2) lymphatic metastasis, 3) hematogenous spread, and 4) transtubal implantation [7]. Of these, metastasis of microinvasive squamous cell carcinoma without lymphovascular space involvement generally develops in lymph nodes [3-6]. In patients with squamous cell carcinoma with a stromal invasion of ≤3 mm, lymph node metastasis was observed in 1.2% [4]. A previous study reported that none of 140 patients with stromal invasion ≤1 mm suffered disease recurrence [8]. However, a patient with a stromal invasion of 0.8 mm who developed pelvic and para-aortic nodal metastases has been reported [3]. It is unlikely that the pelvic tumor in our case developed through lymphatic spread because 1) lymphovascular involvement was not detected in the specimen of conization; 2) no pelvic lymph node swelling was observed at the time of recurrence; 3) the tumor was located outside of the pelvic lymph node area; and 4) the solid mass did not have histopathologic findings of a lymph node structure.

Ovarian metastasis may develop in patients with microinvasive cervical carcinoma through transtubal dissemination [9]. Of note, ovarian metastasis develops almost exclusively in premenopausal women in microinvasive adenocarcinoma and adenosquamous carcinoma [10,11] and adenocarcinoma in situ [12,13]. In contrast, ovarian metastasis in cervical intraepithelial neoplasia and squamous cell carcinoma generally develop in postmenopausal women without periodic endometrial shedding [14,15]. In young women with microinvasive squamous cell carcinoma without lymphovascular space involvement, neither ovarian nor peritoneal recurrence has been reported after cervical conization, to the author’s knowledge. 

In our case, retrograde menstruation appears to be involved in the development of peritoneal recurrence. During perimenstrual time, blood was found in the peritoneal fluid in 90% of women with patent tubes at laparoscopy [16]. Retrograde bleeding, which appears to be associated with the histogenesis of endometriosis, may result in the transtubal migration of viable endometrial cells that attach and implant in the pelvic cavity. Similarly, neoplastic cells existing in the endocervical canal can spread to the peritoneal cavity on the retrograde flow of menstrual blood, which may result in ovarian metastasis in premenopausal women [10-13]. It is unclear why ovarian metastasis did not develop in our case. 

In patients with microinvasive squamous cell carcinoma, intestinal and ovarian recurrences can develop after hysterectomy through an implantation mechanism [17-19]. In these cases, tumor cells appear to exfoliate during a surgical procedure and implant on the surface of the intestine and ovary. Squamous neoplastic cells shed from cervical tumors may grow slowly, as these patients developed late recurrences, 13 and 5 years after hysterectomy, respectively [17,19]. In women with cervical carcinoma treated with minimally invasive surgery, implantation metastasis is known to develop more often than open surgery [20]. 

This case indicates that the peritoneal recurrence of squamous cell carcinoma that developed in a non-irradiated area appears to be radiosensitive and cured with radiation. A complete response was achieved by concurrent chemoradiotherapy, although she had a bulky tumor. 

## Conclusions

Peritoneal recurrence of microinvasive cervical squamous cell carcinoma can develop after conization. Further research is warranted to elucidate the mechanisms and risk factors of peritoneal recurrence in cervical carcinoma.
